# Forecasting emergency medicine reserve demand with a novel decomposition-ensemble methodology

**DOI:** 10.1007/s40747-021-00289-x

**Published:** 2021-03-02

**Authors:** Li Jiang-ning, Shi Xian-liang, Huang An-qiang, He Ze-fang, Kang Yu-xuan, Li Dong

**Affiliations:** 1 School of Economics and Management, Beijing Jiaotong University, Beijing, 100044 China; 2 National Medical Products Administration of China, Beijing, 100037 China; 3 Beijing Wuzi University, Beijing, 101499 China; 4 University of Liverpool, Liverpool, L69 3BX UK

**Keywords:** ARIMA, ELMAN, EMD, Medicine reserve, Public health events

## Abstract

Accurate prediction is a fundamental and leading work of the emergency medicine reserve management. Given that the emergency medicine reserve demand is affected by various factors during the public health events and thus the observed data are composed of different but hard-to-distinguish components, the traditional demand forecasting method is not competent for this case. To bridge this gap, this paper proposes the EMD-ELMAN-ARIMA (ELA) model which first utilizes Empirical Mode Decomposition (EMD) to decompose the original series into various components. The Elman neural network and ARIMA models are employed to forecast the identified components and the final forecast values are generated by integrating the individual component predictions. For the purpose of validation, an empirical study is carried out based on the influenza data of Beijing from 2014 to 2018. The results clearly show the superiority of the proposed ELA algorithm over its two rivals including the ARIMA and ELMAN models.

## Introduction

Given that the emergency medicine reserve is one of the strategic materials to deal with large-scale public health events, emergency medicine reserve management plays an important roles in building up the emergency response capacity. Intuitively, in case of a public health event, the lack of emergency medicines probably wastes valuable rescue time and results in much more loss. For example, at the end of 2017 when the influenza virus was rapidly spreading in several regions of China, the specific medicine “Duffy” was out of stock due to the unexpected demand surge, e.g., a shortfall of 310,000 boxes of "Duffy" only in Beijing. However, overstocking emergency medicines also leads to some major problems like the great stock cost and huge expiration cost. Only with the medicine reserve decision based on the accurate demand forecast, can the above problems be effectively solved. However, the emergency medicine reserve demand is affected by the mixed effects of very complex factors like seasonality, response speed, virus transmission rate (in case of an epidemic), communication network of the people, among others. Consequently, the observed data are composed of different but hard-to-distinguish components; therefore, the traditional demand forecasting method does not work for this case, which presents China Food and Drugs Administration (CFDA) an urgent challenge to optimize the safety stock of emergency medicine reserve. Motivated by the above theoretical as well as practical concerns, this work aims to propose a new forecast algorithm of more accuracy based on the decomposition-ensemble methodology.

The rest of this paper is organized as follows: “Literature review” reviews the latest studies on the medicine demand forecasting; “Problem description” systematically expounds the problems and corresponding methods; “Model” elaborates the EMD-ELMAN-ARIMA (ELA) model; “Empirical study” concludes this paper and makes a discuss on the future studies.

## Literature review

The most widely applied paradigm of forecasting the medicine demand is composed of two phases. The first phase predicts the number of infected cases (persons) while the second generates the forecast of the quantity of medicine demand based on the outcome in the first phase. In terms of forecasting the number of the infected cases, system dynamics models and statistical models are the two mainstream approaches to predict the number of patients in public health events. The most typical system dynamics model is the classic SIR model proposed by Kermack and McKendrick, which lays the foundation of infectious disease dynamics [1]. Since then, infectious disease dynamics models have experienced a rapid development, many variants have emerged, like SEIR model considering epidemic latency [2], SIQR model considering isolation term [3], etc. System dynamics models considered the evolution law and transmission characteristics of public health events, but the prediction accuracy of the models depends on the predefined epidemic transmission mechanism and the parameter settings. Besides, the dynamics of the parameters are expressed by linear models, which cannot capture the non-linear relationship among factors in the epidemic transmission process. Although some studies have tried to simulate the individual behavior and social network in the system with the help of complex network models, such as the random network, small-world network and scale-free network [4–6], these models do not apply to the large-scale systems subject to high complexity.

Statistical models generally predict the evolution trend of public health events based on the historical observations. For examples, [7–12] used ARIMA model and its variants flexibly to predict the propagation path and the number of COVID-19 cases in various countries in the world, and achieved a satisfactory forecasting accuracy. In the study of influenza incidence prediction, Rao et al. collected the data set of a reported influenza epidemic of hospitalized children in a certain hospital, identified the characteristics of the prevalent influenza virus subtypes in different months, seasons, years, and patients' age, and finally used ARIMA model to make the short-term prediction [13]. Wu et al. constructed a stochastic forest regression model for predicting the weekly incidence of influenza-like illness [14]. Furthermore, Tapak et al. made a comparative evaluation of different models for predicting influenza outbreaks, the result showed that neural network was better in outbreaks detection, and time series models had promising performances [15]. In the study of forecasting the incidence of hepatitis B, Wang et al. found that ARIMA model showed better forecasting performance than GM(1,1) model [16]. Zheng et al. showed that ELMAN model was superior to ARIMA (0,1,1) model in predicting the incidence of hepatitis B in Guangxi through comparative experiments [17]. In addition, Wei et al. announced that the ARIMA-GRNN mixed model performs better than the ARIMA model and the GRNN model in predicting the incidence of hepatitis [18].

The advantage of the statistical models is that the models include fewer predefined parameters compared with the system dynamics models. However, these kinds of models are heavily dependent on the historical data, which explain why a statistical prediction model shows inconsistent performance in different studies. The “data-driven” nature makes the input data dominate the outcome of the statistical models, namely “garbage in garbage out”. To provide the suited input data, it is necessary to decompose the original data into different components and select the statistical model according to the component characteristics. Empirical Mode Decomposition (EMD) provides a way to solve this problem. EMD is an analysis and processing method for non-linear and non-stationary signals [19] and has been successfully applied in a wide range of fields, such as energy, finance, agricultural products, hydrometeorology, mechanical fault diagnosis, and so on [20–28]. However, the application of EMD has not been found in predicting the medicine reserve demand in public health events.

There are some studies presenting the whole forecasting process, very few though. These scanty studies include, Guo et al. used the BP neural network method to predict the population of wounded and deaths in the Yushu earthquakes, and then used the safety stock theory to estimate the demand for emergency supplies [29]. Zhang et al. constructed a grey Verhulst prediction model to predict the number of casualties in the earthquakes, and based on average use requirements to determine medical supplies [30]. In addition, Wang further explored a continuous interval gray Verhulst model to work out the demand for emergency medicines in a same mechanism [31]. It can be seen that the existing research only focuses on the immediate demand after the disaster event, and the long-term reserve demand forecast of special emergency medicines for treating public health events has not been found yet.

The current related studies on emergency medicine reserve demand forecasting present 4 characteristics: (1) a majority of studies are focused on forecasting the number of cases, only few studies present a complete solution to forecasting the emergency medicine reserve demand. (2) Compared with the system dynamics model, the statistical model need much less predefined parameters and can capture the development trend in the data-driven way. (3) The statistical models like ARIMA and neural network have been widely used in the research of medicine demand forecasting. However, the performance is sample-sensitive, namely the forecasting accuracy values are different on different samples. (4) Empirical Mode Decomposition has widely been applied to improve the calculation accuracy of the single prediction models in various fields.

Based on the above-mentioned, this paper proposes the EMD-ELMAN-ARIMA (ELA) hybrid forecasting model. ELA uses EMD method to decompose the historical data of the infected cases into different components, then ARIMA and ELMAN neural networks are employed to capture the linear and the non-linear components, respectively. Furthermore, based on the projection from the number of the infected cases to the medicine reserve demand dominated by nature of the public health events (i.e., the average medicine demand per person during the public health event), the emergency medicine reserve demand can be obtained. Finally, to validate the model, an empirical study is carried out based on the actual data of influenza cases during the period from 2014 to 2018 in Beijing. To the best of our knowledge, this paper is the first to apply EMD to the emergency medicine reserve demand forecasting.

## Problem description

A. Characteristics of emergency medicine demand

Before forecasting the emergency medicines reserve demand, it is necessary to identify the characteristics of the data so as to select the matching forecasting methods. The demand for emergency medicines is characterized by suddenness, surge, and urgency. First, it is difficult to foresee the outbreaks of public health events, especially to accurately predict its infection scope and population, so the need for emergency medicine that will take on a sudden character. Second, the denser population and complex movement of people within cities accelerate the spread of epidemic, with the spread of online public opinion, the sales of emergency medicines will show a stronger surge character. Finally, the needs of emergency medicines are also extremely urgent, as the availability of emergency medicines cannot be delayed after the disease outbreak, the lack of emergency medicines reserve tends to fuel social panic, which can lead to more serious social problems.

In combination with the above characteristics, it is easy to infer that the data on the number of people affected by public health events are destined to be strongly non-stable, so the traditional time series prediction method, which requires a high requirement for data stationarity, will not be too accurate. At the same time, traditional regression analysis methods are difficult to apply to such issues because of the difficulty of identifying and quantifying the factors that lead to the surge in demand for emergency medicines. In addition, considering the rapid population movements, frequent trade and commerce, and rapid development of the cities, premature demand data do not provide much guidance for the current forecast results. Therefore, models of emergency medicine reserve requirements also require the ability to handle small sample data. To sum up, forecasting the demand for emergency medicines is not only a hot academic issue, but also a very difficult practical issue.

B. Paradigm of emergency medicine reserve demand forecasting

**Fig. 1:**
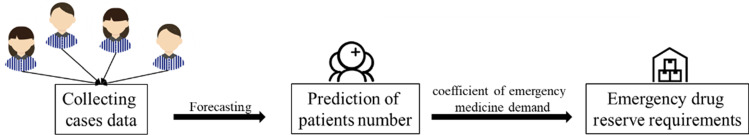
Paradigm of emergency medicine reserve demand forecasting

In practice of CFDA, the medicine reserve demand is estimated by multiplying the number of suspected cases by the effective infections coefficient (i.e., the average medicine demand per person). Therefore, this paper proposes a paradigm of emergency medicine reserve demand forecasting (see Fig. 1).

The proposed paradigm proceeds as follows: First, historical data are collected on the actual number of people suffering from public health events in cities. Second, historical data are used to forecast the specific number of people suffering from diseases and the trend over the coming period. Finally, based on the prediction of the patients, the demand for emergency medicine reserve is estimated according to the medicine production capacity, reserve demand characteristics, as well as the guidance and recommendations of experts, all the mechanisms and relevant factors can be summarized as the demand coefficient of emergency medicine $\alpha$. Denote $S$ the reserve demand of emergency medicines and *p* the predicted value of cases in a public health event, the emergency medicines reserve demand can be expressed as follows:

1 $$ S = p \times \alpha $$

The demand coefficient $\alpha$ of treatment medicines is usually equal to 1, that is, one patient corresponds to one person's medicine demand. However, in the real storage situation, the medicine storage units often need to adopt a safer demand coefficient bigger than 1, and their values are not the same according to the operation situation of the medicine storage enterprises, the instructions of the national medicine regulatory authorities, and the medicines production and procurement situation.

## Model

Statistical prediction models can classified into three categories including regression models (e.g., Logistic Regression, Multiple Regression, etc.), time series models (e.g., Exponential Smoothing method, ARIMA, Bayesian VAR, etc.), and artificial intelligence models (e.g., Neural Networks, Support Vector Machines, etc.). In previous studies, the reliable performance of ARIMA and neural network models like ELMAN motivates this paper employ them as individual models.

A. Autoregressive integrated moving average model

The Autoregressive Integrated Moving Average Model (ARIMA) is one of the most frequently used models. The ARIMA model is based on the idea of transforming a non-stable time series into a stable time series $x\left( t \right)$ composed of the auto-regression of $x\left( t \right)$ and the moving average of the random term. The mathematical expression is as follows:

2 $$ x\left( t \right) = c + \emptyset_{1} x_{t - 1} + \emptyset_{2} x_{t - 2} + \ldots + \emptyset_{p} x_{t - p} + \varepsilon_{t} + \theta_{1} \varepsilon_{t - 1} + \theta_{2} \varepsilon_{t - 2} + \ldots + \theta_{q} \varepsilon_{t - q} $$

where $x\left( t \right)$ is the actual number of the cases caused by public health events, $c$ is constant, $\emptyset_{i}$ and $\theta_{i}$ are the coefficients, $p$ and $q$ referred to as autoregressive and moving average, respectively.

B. ELMAN neural network model

**Fig. 2:**
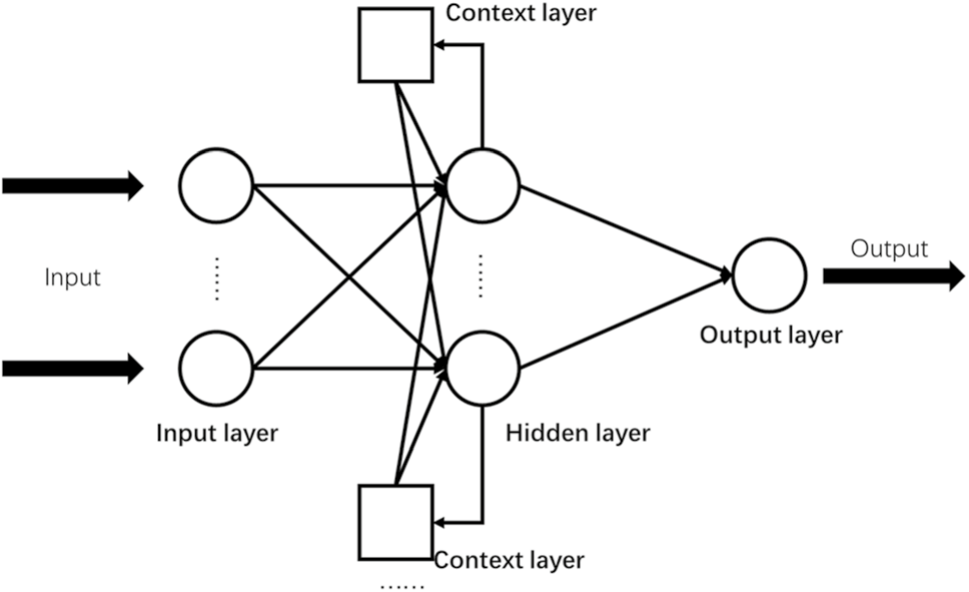
An illustrative Structure of ELMAN Neural Network Model

Neural network models have been proved to be a kind of effective prediction models in a wide range of fields [32–34]. ELMAN neural network is a specific realization of the feedback neural network and shows its power in the prediction of influenza epidemics [15]. The network consists of the input layer, the hidden layer, the context layer, and the output layer. The functions of the input layer, hidden layer and output layer are alike to those of other neuro networks. The context layer acts as a time delay operator in the model, linking the previous output value from the hidden layer to the next input of the hidden layer, forming an internal feedback network. An illustrative structure of ELMAN neural network is shown in Fig. 2.

To describe the non-linear state space of the ELMAN neural network model [35], the definitions can be made as follow: $w^{1} ,w^{2} ,w^{3}$ are the weights of the three steps of the input layer-hidden layer, hidden layer-context layer, and hidden layer-output layer, respectively; $x\left( k \right)$ is the output of the hidden layer and $x_{c} \left( k \right)$ is the output of the context layer; $f\left( \cdot \right)$ and $g\left( \cdot \right)$ are the transfer functions of the hidden layer and the output layer, respectively; $0 < \partial < 1$ indicates the internal feedback gain factor, and the final network output is represented by $y\left( k \right)$. The derivation of the relationship of the model is as follows:

3 $$ y\left( k \right) = g\left( {w^{3} x\left( k \right)} \right) $$

4 $$ x_{c} \left( k \right) = \partial x_{c} \left( {k - 1} \right) + x\left( {k - 1} \right) $$

5 $$ x\left( k \right) = f\left( {w^{1} u\left( {k - 1} \right) + w^{2} x_{c} \left( k \right)} \right) $$

C. Distinguishing components by EMD

Although the above-mentioned models have seen some successful applications in emergency medicine demand forecasting studies [36–39], their performances are affected by the noise in original data in the prediction process. There are many factors that affect the number of people affected by a public health event, for example, the number of people infected by a specific influenza outbreak may be influenced by temperature, moderation, population density, mode of transmission, precautions, and other factors. Therefore, the original time series tends to comprise very complex components, which renders a big challenge for the forecasting task. Despite that it is proved that including the key factors into the model can improve the prediction results [40, 41], it is impossible to identify and quantify all the factors, consequently some useful components cannot be captured by the traditional models and are ignored as noise, which lead to unsatisfactory performance of the traditional models.

To overcome the above problem, inspired by the studies [42, 43], this paper uses Empirical Modal Decomposition method (EMD) to decompose the original data. EMD is an adaptive method suitable for processing non-stationary and non-linear series. EMD is totally data-driven and enjoys advantages compared to wavelet decomposition owing to its non-parametric nature. Out of the original data, a set of Intrinsic Mode Functions (IMFs) can be extracted, which provides more insight into the dynamics of the data and facilitates the forecasting work.

D. The EMD-ELMAN-ARIMA (ELA) model

**Fig. 3:**
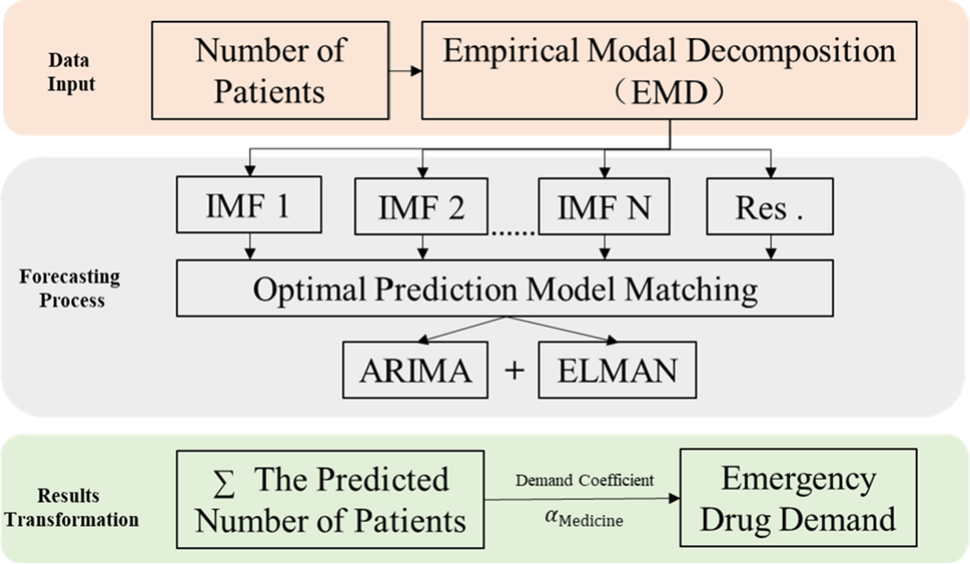
The model of EMD-ELMAN-ARIMA

It is a unanimous agreement in the forecasting field that the models, including time series models, econometric models and artificial intelligence methods, should be carefully tailored to fit the data characteristics. Given that the emergency medicine demand against the public healthy events comprise multiple components of different characteristics, a single model is not sufficient. Considering some studies have shown the power of combined forecasting models in this case [44, 45], this paper proposes the combined model, namely ELA, based on the decomposition-ensemble methodology, which proceeds as follows: First, the time series of the cases (patients) is decomposed into different components by EMD. Second, ARIMA and ELMAN neural network are employed to predict the components, based on which the integrated outcome is generated to be the sum of the forecasts of the components; finally, the prediction of emergency medicines reserve demand is obtained by Eq. (1). Figure 3 visually depicts the proposed ELA model.

## Empirical study

A. Data introduction

With the support of CDFA, we obtained the weekly data on historical influenza cases from Beijing Center for Disease Prevention and Control (Beijing CDC) during the period of 01/07/2014 to 25/12/2018. The first 200 observations are used to tune the parameters of the models, and the remainder 21 observations from August to December 2018 are used to validate the model.

**Fig. 4:**
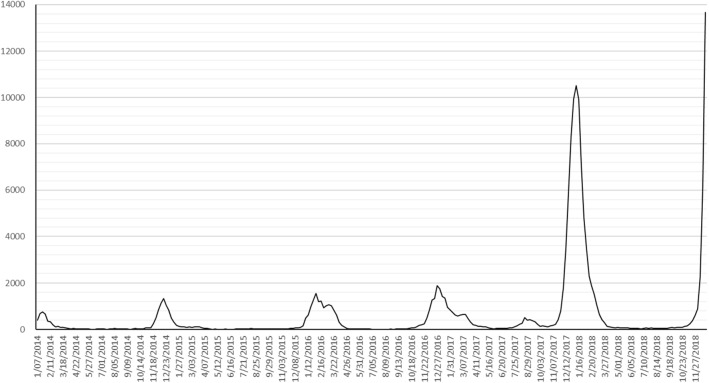
Original data of influenza cases

Figure 4 visualizes the original data and shows a significant increase of the peak level of the reported cases after 2018. According to the experts from Beijing CDC, the main reasons are as follows: First, at the end of 2017, a new influenza virus subtype appeared in Beijing, and the population did not have immunity to this new virus subtype, resulting in an increase in susceptible populations; Second, due to the previous media propaganda, including the posts of influenza deaths reprinted on the Internet, the public's awareness of the dangers of influenza and the awareness of vigilance have increased significantly, resulting in a significant increase in the rate of medical treatment and the number of reported cases; Third, a new detection technology has been widely used in medical institutions in Beijing since 2017, and the detection rate and detection rate have been improved. The superposition of the three factors led to a significant increase in the number of influenza cases reported, but it still did not reach the outbreak level.

B. Data processing

Due to the complexity of influenza in megacities, the cases of influenza patients tend to be highly volatile and unstable. Using the Augmented Dickey–Fuller test, the stationarity of the data can be tested. Table 1 lists the test results showing that the original series is not stationary, which implies that the models for stationary series cannot be applied in this case.

**Table 1:** Stationary test of influenza cases data

Augmented Dickey-Fuller test statistic		*t* Statistic	Prob.*
		− 0.229754	0.9920
Test critical values:	1% level	− 4.003449	–
	5% level	− 3.431896	–
	10% level	− 3.139664	–

**Fig. 5:**
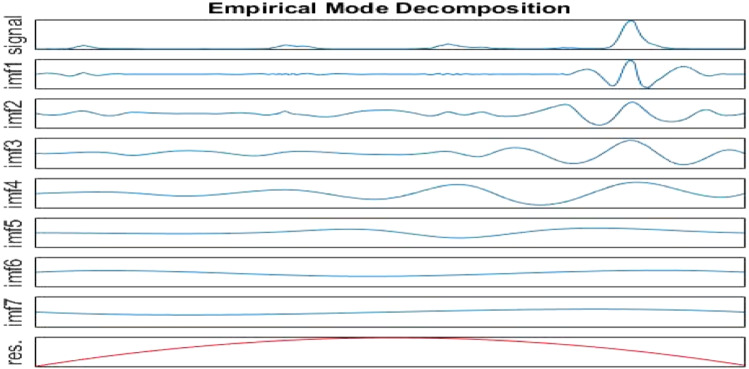
IMFs generated by EMD

**Fig. 6:**
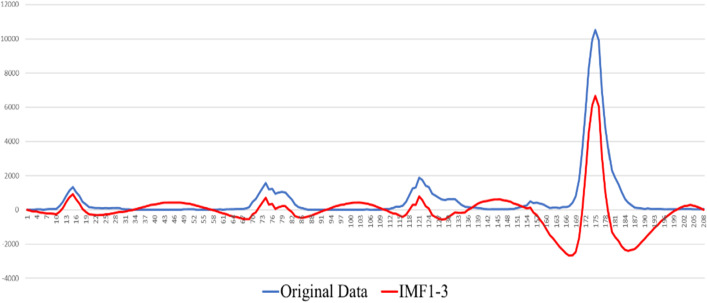
Original time series V.S. modes of IMF1-3

**Fig. 7:**
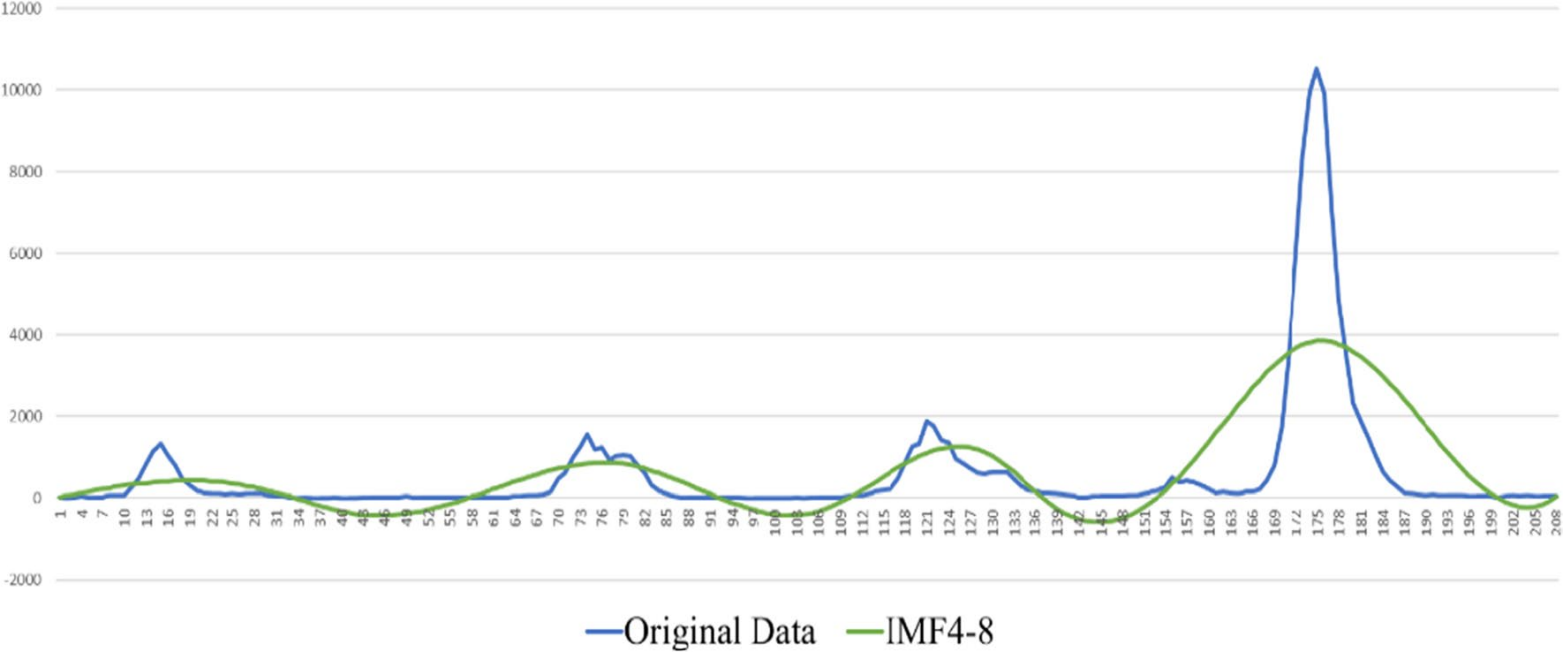
Original time series V.S. modes of IMF4-Res

This section uses the proposed ELA model to predict the influenza cases. First, the data were decomposed by EMD using the MATLAB software package. As shown in Figs. 5, 6, 7, IMFs and one residual mode were obtained after EMD decomposition. The IMF1 has the highest frequency and fluctuating most strongly at random. The frequency decreases when we check the modes from IMF2 to the residual series. As shown by [40, 41], data preprocessing plays a very important role in forecasting. For higher forecasting accuracy, it is necessary to appropriately preprocess the data before run the forecasting models, among which, matching the IMFs, individually or in combinations, with the suited models is a key task.

We tested various combinations of 8 modes by “trial and error”. According to the results, the sum of IMF1 to IMF3, denoted by $x_{1}$, fits best the influenza cases data. While the sum of IMF4 to the residual series, denoted by $x_{2}$, simulates well the seasonality. Thus, the series of influenza cases is decomposed into two subseries $x_{1}$ and $x_{2}$, and the original series $x$ of influenza cases in Beijing can be expressed as Eq. (6).

6 $$ x = x_{1} + x_{2} $$

**Fig. 8:**
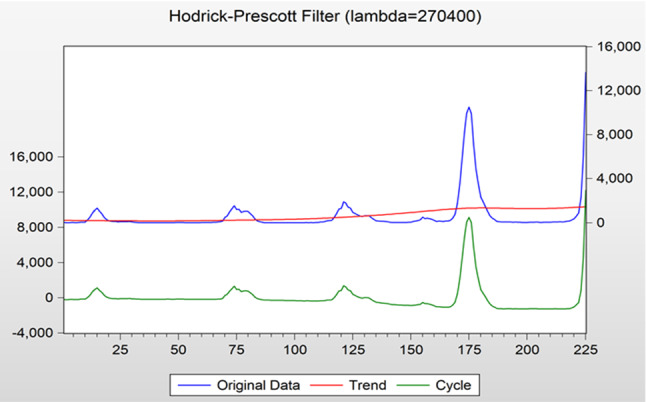
Decomposition of original time series by hodrick-prescott filter

**Fig. 9:**
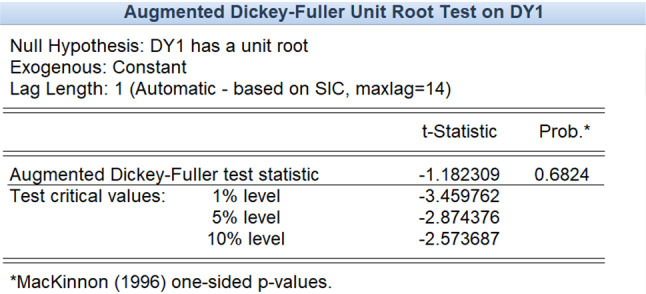
ADF test results of first-order difference in the original time series

**Fig. 10:**
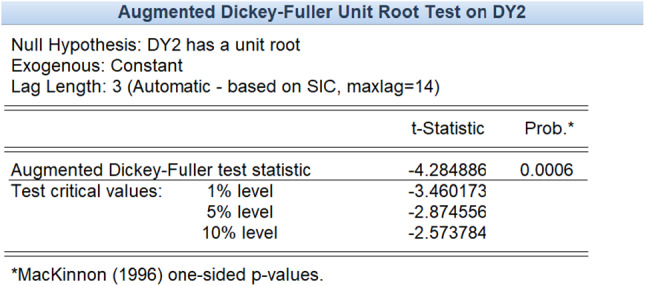
ADF test results of second-order difference in the original time series

**Fig. 11:**
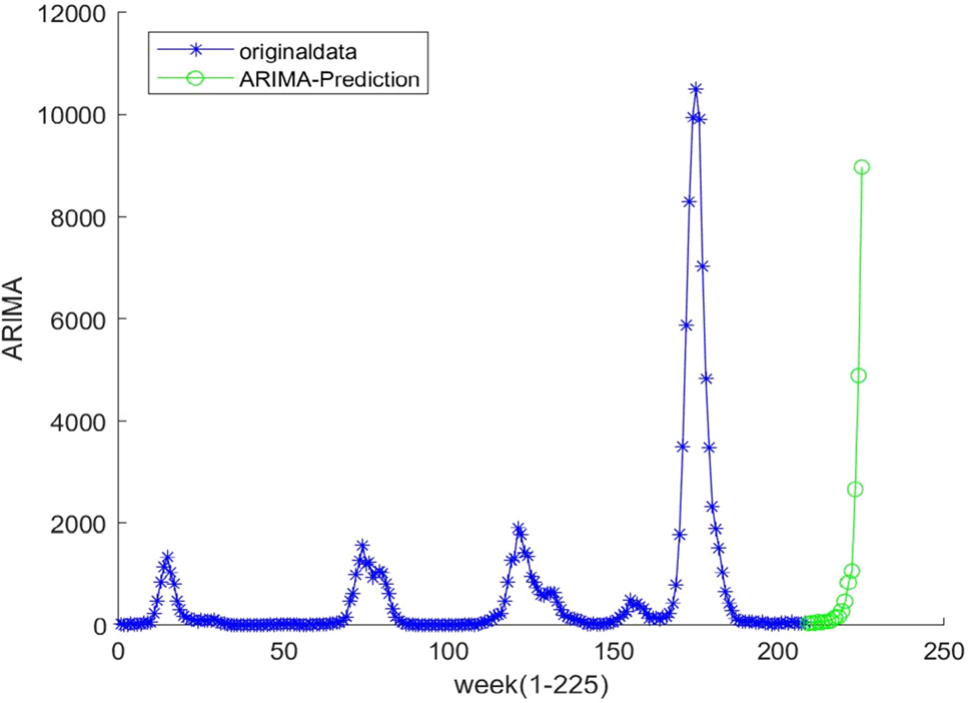
Fitting results of ARIMA(0,2,4) model

The ELMAN neural network and ARIMA models are used to capture the nonlinearity in × 1 and seasonality in × 2, respectively (Figs. 8, 9, 10, 11).

C. Forecasting process

The whole forecasting process is composed of 4 steps. First, ARIMA model is used to predict the original time series. Because the data fluctuate excessively and include the growth trend brought by the progress of influenza virus detection technology, it is necessary to strip the trend items brought by the progress of detection technology from the original data before using ARIMA model. With the help of Hodrick–Prescott filtering method, the original time series is divided into the period term and trend term, and the trend term is added to ARIMA model as an explanatory variable for model fitting [46].

Because the original data fluctuate violently, the original time series is processed by difference, and the first-order and second-order difference series are tested by the ADF test.

As shown in the above test results, at the significance level of 0.05, the null hypothesis is rejected, indicating that the second-order difference series of the original influenza cases data is stationary. The Box–Jenkins method is used to determine the order of the model, and the ARIMA (0,2,4) model is obtained on the premise that the residual error sequence is a white noise sequence.

**Fig. 12:**
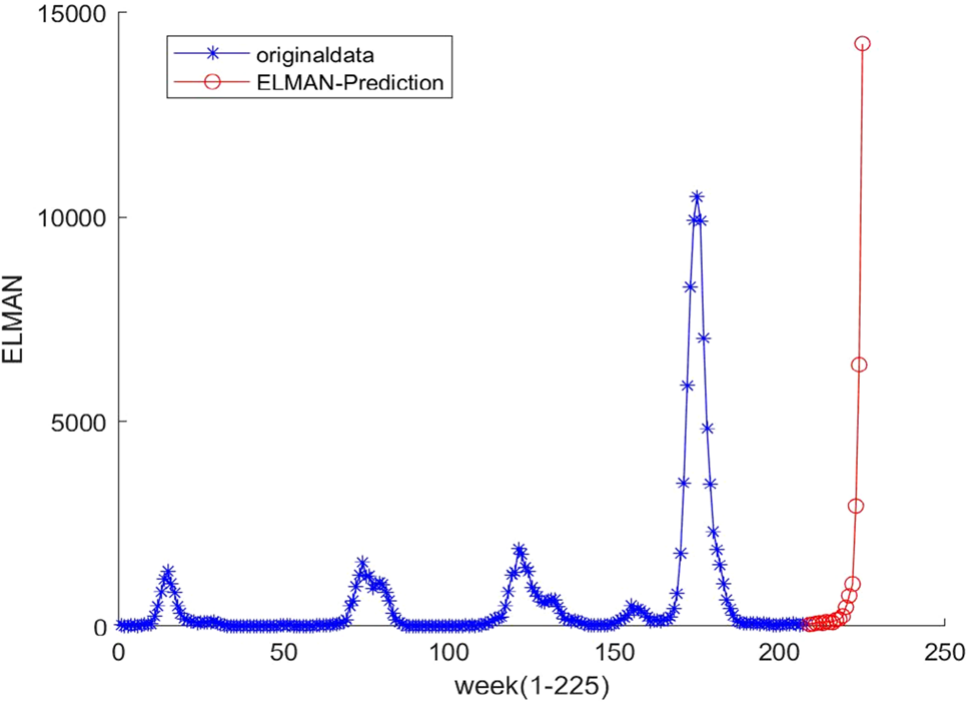
The ELMAN model predicts results

Second, in the ELMAN neural network, we used the training data set (the blue points in Fig. 12) to estimate the network parameters, based on which we obtained the forecasts (the red points in Fig. 12).

Third, the ELA model was used to predict the number of cases given the two combinations of IMFs, i.e.,${ }x_{1}$ and $x_{2}$. The ELMAN neuro network was used to get the forecasted value of ${ }x_{1}$. Using the AIC rule, ARIMA (4,1,1) was identified to be the best fit for $x_{2}$. The final predicted values were obtained by summing up 21 predicted values of the $x_{1}$ and $x_{2}$ in the forecasting horizon from 07/08/2018 to 25/12/2018.

**Fig. 13:**
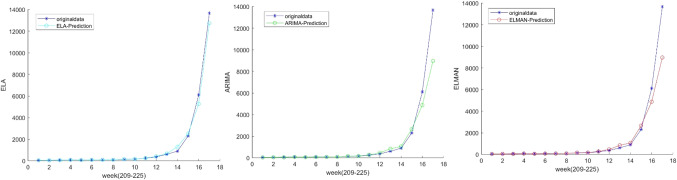
The comparison of three prediction methods

Fourth, after obtaining the prediction results of the number of influenza patients, it is necessary to estimate the projection coefficient $\alpha$ from the number of cases to the emergency medicine reserve demand. According to the experts from CDFA and Beijing CDC, $\alpha$ is dependent on the treatment course and dosage, and $\alpha = 1$ would be acceptable by rule of thumb in case of the influenza (Fig. 13).

D. Prediction results and analysis

In this paper, Mean Absolute Percentage Error (MAPE) is used to evaluate the prediction error. Denote $\widehat{{x_{t} }}$ the predicted value and $x_{t}$ the data of the original time series, then MAPE is mathematically expressed as follows:

7 $$ MAPE = \frac{1}{n}\mathop \sum \limits_{t = 1}^{n} \left| {\frac{{\widehat{{x_{t} }} - x_{t} }}{{x_{t} }}} \right| $$

On the one hand, for the prediction results of the number of influenza patients, the combined prediction model constructed in this paper is superior to the traditional time series model and machine learning model alone.

According to Table 2, the forecasting error of the ARIMA models reaches 15.31% and 10.62%, the ELMAN neuro network suffers a forecasting error of 7.29%, while the proposed ELA model sees a significantly smaller error of 3.87%, which implies that the ELA model outperforms the individual ARIMA and ELMAN models. With $\alpha = 1$, we obtained the predicted value of the emergency treatment medicines for influenza in 2018 as shown in Table 3:

**Table 2:** Performance comparison of different models

Model	MAPE (%)
ARIMA-original	15.31
ARIMA-dummy	10.62
ELMAN	7.29
ELA	3.87

**Table 3:** Model performance Comparison based on Actual Reserve in 2018

Method	Forecasts	Actual medicine reserves for influenza
ARIMA	75,193	78,940
ELMAN	82,210	
ELA	79,283	
Number of real cases	80,247	

Table 3 shows that the forecast by ELA fits best the actual medicine reserve demand, compared with its 3 rivals. It is worth noting that the actual medicine reserve for influenza in 2018 is less than the forecast, which is consistent with the reality according to the administrative of CDFA, saying there was a shortage of the medicine reserve for influenza and China started the emergency production at a higher cost than in usual. The relief action would have been more effective if the demand was estimated accurately, which highlights the practical value of the ELA model.

## Conclusion

Based on the decomposition-ensemble methodology, this paper constructs a new combined prediction model based on EMD, ARIMA, and ELMAN, namely the ELA model, to forecast the emergency medicine reserve demand in response to a public health events. This model employs EMD decompose the original series of cases (patients) into different components, then reconstructs all the components into several combinations to avoid the curse of dimensionality. Subsequently ELMAN neuro network and ARIMA are applied to the suited combinations by “trial and error” according to the characteristics, like linearity and nonlinearity in this study. Finally, the forecast of the emergency medicine reserve demand is generated with a projection coefficient $\alpha$ by the rule of thumb. To validate the model, with the support of CFDA and Beijing CDC, an empirical study was carried out based on the weekly data of influenza cases in Beijing from 07/08/2018 to 25/12/2018. The results clearly show the superiority of the proposed ELA model over its rivals, which indicates the potential of the ELA model to be a more powerful tool for emergency medicine reserve management.

It is notable that although this work promotes better understanding of applying the decomposition-ensemble paradigm to the emergency medicine reserve forecast work, some important issues are left to the future studies. For examples, when EMD generates a large scale of components, it is infeasible to identify the best combinations by “trial and error”. Moreover, there are some optimization problems to be solved in the future, like the optimal length of forecasting horizon, the optimal number of individual models to be integrated, the optimal size of training set, and so forth.
